# Genomic, Proteomic, and Biochemical Study of *Pleurotus pulmonarius* Secretome and Its Role in Biomass Saccharification

**DOI:** 10.1021/acs.jafc.5c09170

**Published:** 2025-10-27

**Authors:** Romanos Siaperas, Eftychia Papadaki, Panagiotis Giannikos, Anastasia Zerva, Evangelos Topakas

**Affiliations:** † Industrial Biotechnology & Biocatalysis Group, Biotechnology Laboratory, School of Chemical Engineering, 68994 National Technical University of Athens, 9 Iroon Polytechniou Street, Attiki, Athens 15772, Greece; ‡ Laboratory of Enzyme Technology, Department of Biotechnology, School of Applied Biology and Biotechnology, 68995 Agricultural University of Athens, 75 Iera Odos Street, Attiki, Athens 11855, Greece; § Biochemical and Chemical Process Engineering, Division of Sustainable Process Engineering, Department of Civil, Environmental and Natural Resources Engineering, Luleå University of Technology, Luleå 971 87, Sweden

**Keywords:** Pleurotus pulmonarius, genomics, proteomics, secretome, corn stover, beechwood, lignocellulose, CAZymes, synergism, biorefinery, saccharification

## Abstract

*Pleurotus* species include edible mushrooms that grow on a range of lignocellulosic substrates by secreting hydrolytic and oxidative enzymes. This flexibility offers opportunities to valorize agro-industrial residues for sustainable production of food and biomaterials. In this study, we analyzed the genome of *Pleurotus pulmonarius* and its biocatalytic potential for lignocellulose saccharification using proteomics and biochemical assays. The fungus was cultivated on beechwood, corn stover, and xylose. Beechwood induced the richest secretome with abundant oxidases. The corn stover secretome had fewer proteins but was focused on carbohydrate-acting enzymes, with abundant polysaccharide-degrading and accessory enzymes. Despite lower enzyme diversity, corn stover secretome achieved higher lignocellulose saccharification, further improved by oxidoreductase inhibition. Supplementing an industrial cellulase cocktail with *P. pulmonarius* corn stover secretomes enhanced sugar release by 40%. These findings highlight the dynamic enzymatic response of *P. pulmonarius* to lignocellulosic substrates and its potential in biomass valorization and the design of enzymatic cocktails.

## Graphical abstract

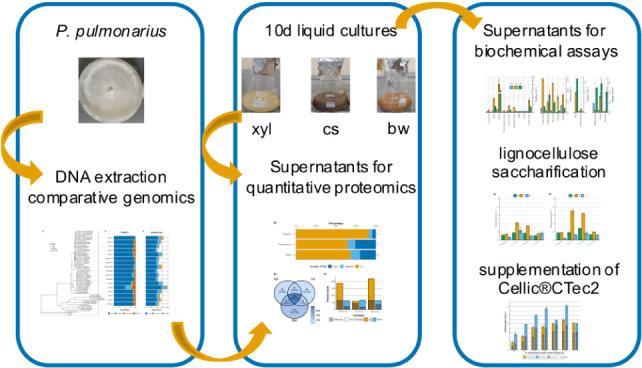

## Introduction

Biomass of terrestrial plants represents the most abundant source of biobased carbon on Earth, accounting for 80% of the estimated 550 gigatons.^1^ Most of this biomass is concentrated in the plant cell wall, composed primarily of cellulose, lignin, and hemicelluloses, along with pectin and other biopolymers.^2^ Lignocellulosic biomass (LCB) is thus a promising feedstock for the transition from petroleum to biobased refineries. However, effective utilization of LCB remains challenging due to its recalcitrance to degradation and saccharification. Lignin is one of the key factors for this recalcitrance, being a complex aromatic polymer, which is cross-linked with hemicellulose and is crucial for structural integrity, waterproofs the cell wall and hinders enzyme access to the polysaccharide-rich matrix.^3,4^

Although some bacterial species are capable of partly oxidizing lignin,^5^ the primary lignin degraders in nature are white-rot fungi (WRF) belonging to the Agaricomycotina subphylum.^6^ These filamentous fungi secrete a cocktail of hydrolytic (cellulases and hemicellulases) and oxidative (ligninolytic and accessory) enzymes, along with noncatalytic carbohydrate-binding modules.^7^ Together, these proteins enable efficient degradation of all LCB constituents.^8^ The enzymes responsible are known as carbohydrate-active enzymes (CAZymes) and are classified in the CAZy database alongside associated binding modules.^9^ Building on CAZy, dbCAN was developed,^10^ which uses profile Hidden Markov Models (HMMs) for automated CAZyme annotation, subfamily classification, and enzyme commission (EC) assignment.

The genus *Pleurotus*, within the Agaricales order of WRF, includes edible, ligninolytic mushrooms with widespread use in bioremediation, agriculture and food industry.^11–14^ Members of *Pleurotus* genus can grow on a broad range of lignocellulosic substrates, including both woody and nonwoody biomass.^15,16^ This flexibility offers opportunities to valorize agro-industrial residues for sustainable production of food, bioactive compounds and biomaterials.^7,17–19^

Advances in high-resolution, high-throughput mass spectrometry-based proteomics^20^ have enabled comprehensive analysis of fungal secretomes, uncovering the presence, abundance, and post-translational modifications of the secreted proteins. Despite the potential of *Pleurotus* species, proteomic data sets are currently limited to *P. ostreatus* ^13,15,21^ and *P. eryngii*.^16,22^ In addition, proteomic studies in WRF will enhance the design and development of industrial enzyme cocktails, which are currently dominated by ascomycetous enzymes.^23^ By comparison, studies on WRF secretomes report poor to moderate performance in regard to biomass saccharification, compared to ascomycetes.^24,25^

In this study, we analyzed the genome of *P. pulmonarius* LGAM 28684 (formerly misidentified as *P. citrinopileatus*) and investigated its biocatalytic potential for saccharification of hydrothermally pretreated LCB using LC-MS/MS proteomics and biochemical assays. This strain has previously been studied for its high laccase production^26^ and its ability to treat phenolic-rich olive oil mill wastewater.^12^ Here, we show that this strain secretes a rich arsenal of lignocellulose-degrading enzymes, tailored to the growth substrate, with emphasis on hydrolases for the degradation of recalcitrant corn xylan. We further demonstrate the synergism of *P. pulmonarius* culture supernatants with commercial cellulases, pointing to a promising system for next-generation enzyme cocktail development and biorefinery applications.

## Materials and Methods

### Materials, Enzymes, Substrates, and Microorganisms

Chemicals and materials are described in Text S1.^27,28^ The *Pleurotus pulmonarius* LGAM 28684 strain was obtained from the culture collection of the Laboratory of General and Agricultural Microbiology (Agricultural University of Athens).

### Genome Sequencing, Assembly, and Annotation

DNA isolation, sequencing, genome assembly, and repeat identification were performed as described in Taxeidis et al.^29^ The structural and functional annotation is described in Text S1.^30–36^

### Orthology Inference and Phylogeny of *Pleurotus*

We performed a comparative analysis of the *Pleurotus* genus using all available proteomes from UniProt^37^ and GenBank. When only non-annotated genomes were available for a species, protein-coding genes were predicted using GeneMark-ES v4.71.^33^ Gene families and single-copy orthologs were identified using OrthoFinder v3.0.1b1.^38^

In order to resolve the taxonomy of*P. pulmonarius* LGAM 28684, a phylogenetic analysis of the *Pleurotus* genus was performed using data from a phylogenetic analysis of the*P. ostreatus* species complex based on 40 nuclear single-copy orthologous genes^39^ as described in Text S1.^40–43^

### Cultivation of *P. pulmonarius* for Secretome Analysis

Cultures with xylose, beechwood and corn stover and secretome preparations are described in Text S1. Fungal biomass was first grown in 100 mL precultures with xylose as the sole carbon source for 10 days.

### Proteomic Analysis

Samples included three replicates for each condition and were processed in two batches at the VIB Proteomics Core (Ghent, Belgium) for label-free LC-MS/MS analysis. The first batch included one replicate of corn stover and two replicates of xylose, with the remaining replicates corresponding to the second batch. Sample preparation, peptide separation and MS/MS data acquisition are described in Text S1. The mass spectrometers were operated in positive ion data-dependent acquisition (DDA) mode and the instrument performance was monitored using Qcloud.^44^ Raw files were searched together using the nf-core/quantms v1.3.1dev pipeline^45,46^ and quantitative analysis was performed with the prolfqua R package.^47^ Detailed methodology of the data processing is presented in Text S1.^48–55^

### Enzyme Activities and Synergism Assays

Enzyme assays on isolated polysaccharides, model substrates and lignocellulosic biomass along with the synergism assays with commercial cellulases are described in Text S1.^26,56–58^

## Results and Discussion

### Genome Assembly and Annotation

In this study, we investigated the lignocellulolytic potential of *P. pulmonarius*LGAM 28684 using high-throughput proteomics and biochemical assays. To enable a comprehensive omics-level analysis, we sequenced, assembled, and annotated its genome. DNA extracted from cells grown in liquid cultures was sequenced with Illumina NovaSeq X Plus, yielding a total of 5.14 Gb. K-mer analysis of the raw reads with GenomeScope estimated a 2.32% heterozygosity rate and revealed a k-mer spectrum characteristic of a heterozygous diploid genome,^59^ with distinct peaks at ∼40× and ∼80× coverage representing heterozygous and homozygous regions, respectively (Figure S1). We assembled the genome with the Redundans pipeline, incorporating the Platanus assembler,^60^ optimized for heterozygous genomes.^61^ The final assembly comprised 2,651 scaffolds with a size of 41.1 Mb and showed high fragmentation due to short-read limitations.

We performed structural annotation with the BRAKER3 pipeline, using RNAseq data from another*P. pulmonarius* strain^31^ and the fungal proteins of OrthoDB v11 ^34^ for training of gene finding algorithms. The RNAseq data were chosen after screening the Sequence Read Archive (SRA) data and had a mapping rate of 87.42% to the LGAM 28684 genome. The annotation identified 14,485 protein-coding genes, encoding 15,491 proteins including isoforms, with 94.9% containing both start and stop codons. We predicted protein function for 8,573 genes using multiple databases and stringent thresholds (see Methods). However, 4,413 genes (30.47%) encoded proteins of unknown function, lacking known domain or motifs. Only 335 genes had significant alignments to SwissProt entries based on the criteria of Haas et al.,^36^ underscoring the limited characterization of *Pleurotus* proteins.

### CAZyme Annotation

To assess the potential of *P. pulmonarius* LGAM 28684 for lignocellulose degradation, we prioritized the annotation of carbohydrate-active enzymes (CAZymes). We used the dbCAN pipeline, replacing its default CAZy FASTA file with an updated version including fungal CAZymes from the Mycocosm database.^62^ dbCAN integrates three databases and developers recommend filtering for proteins supported by at least two of them for automatic genome annotation. To recover additional true positives, we manually reviewed single-database hits. All proteins annotated by either HMM-based method were retained, adding 8 dbCAN HMM-only and 15 dCAN_sub-only hits. In contrast, DIAMOND-only hits had a high false-positive rate: of 209 candidates, only 22 were retained after manual review, with most discarded hits misclassified as glycoside hydrolases (GH) and glycoside transferases (GT), 56 and 47, respectively. The inclusion of curated single-database hits increased the number of annotated CAZymes by 8.01% relative to the two-database consensus, while the majority (82.69%) was supported by all three databases (Table S1).

### Phylogenetic Analysis

*P. pulmonarius*LGAM 28684 was previously misidentified as*P. citrinopileatus*. To clarify its taxonomy, we performed phylogenetic analysis using 40 gene markers across 66 *Pleurotus* isolates and 2 *Hohenbuehelia* outgroups. We retrieved the marker sequences by a previous study of the *P. ostreatus* species complex from GenBank,^39^ using them as BLAST queries to identify orthologous regions across all genomes included in this study. LGAM 28684 clustered with three Italian isolates of *P. pulmonarius* ^63^ in a well-supported clade, with a sister clade composed of French isolates of *Pleurotus* sp. 7 (Figure 1a).

**Figure 1:**
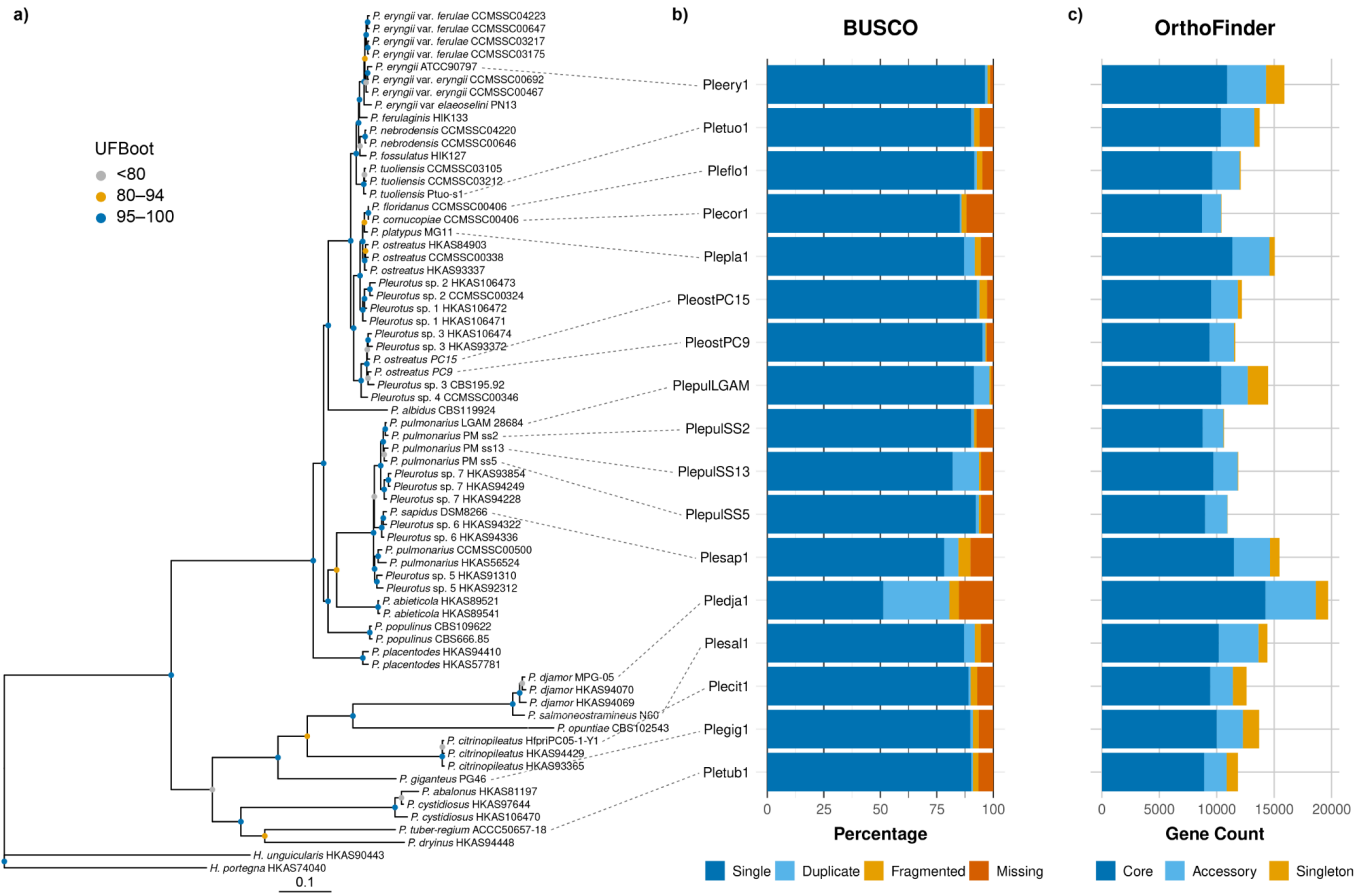
(a) Maximum likelihood phylogeny of 66 *Pleurotus* and two *Hohenbuehelia* isolates inferred from the 40 loci of Li et al.^39^ Nodes are colored based on UFBoot values. Branch lengths correspond to the expected number of substitutions per site. (b) BUSCO completeness of the available *Pleurotus* proteomes using the Agaricales odb_10 data set. (c) Number of protein-coding genes assigned to core (blue), accessory (light blue), and strain-specific (yellow) orthologous groups.

We then conducted a comparative analysis using all available *Pleurotus* proteomes from UniProt and GenBank. For species lacking annotated genomes, protein-coding genes were predicted using GeneMark-ES v4.71 optimized for fungi.^33^ BUSCO^64^ analysis at the proteome level revealed that our predicted proteome was the most complete (98.3%), underscoring the sensitivity of our annotation strategy (Figure 1b). *P. eryngii* and*P. ostreatus* PC9, both downloaded from UniProt, followed with completeness scores of 97.5% and 96.3%, respectively.

### Orthogroups Classification

Using Orthofinder, we grouped genes from 17 *Pleurotus* genomes into 10,799 orthologous groups. Of these, 53.93% formed the core *Pleurotus* panproteome (present in at least 16 genomes), while 37.12% were assigned to the accessory fraction. To assess the impact of lower-quality annotations, we excluded three proteomes with BUSCO completeness below 90% (*P. djamor*, *P. sapidus*, and *P. cornucopiae*). This adjustment slightly increased the proportions of core (56.40%) and accessory (40.48%) groups. We identified 1,203 single-copy orthogroups, a relatively low number given that counts typically increase at lower taxonomic levels, and OrthoDB v10 lists 3,870 single-copy orthologs for the Agaricales order. The reduced count likely reflects high gene duplication rates, particularly in *P. djamor* and *P. pulmonarius* SS2 proteomes (Figure 1b). Removing these raised the number of single-copy orthogroups by 130.42%.

Among all analyzed genomes, *P. pulmonarius* LGAM 28684 had the highest percentage of singleton genes (12.25%), followed by *P. giganteus* (10.26%) and *P. eryngii* (10.08%) (Figure 1c). Most LGAM-specific genes (96.51%) encoded proteins of unknown function, with a median length of just 101 amino acids, much shorter than the proteome-wide median of 343 amino acids. In contrast, the other three *P. pulmonarius* genomes contained fewer than 100 singleton genes and under 12,000 predicted proteins. These differences likely originate from variations in the RNAseq datasets and annotation parameters used with BRAKER by different research groups.^63^ Such methodological inconsistencies are known to negatively affect orthology inference and the detection of lineage-specific genes.^65^

### Proteomic Analysis of the Secretomes

We analyzed the secretomes of *P. pulmonarius* LGAM 28684 grown on xylose and two lignocellulosic substrates, corn stover and beechwood, using label-free, data-dependent LC-MS/MS proteomics in biological triplicates. Out of 520,137 acquired MS/MS spectra, 42.25% were successfully assigned to peptides in the reference protein database, a notably high identification rate given that ∼75% of spectra in typical MS experiments remain unassigned.^66^ Of the identified spectra, 10.5% matched common contaminants, primarily keratins from human skin and hair, and trypsin used for digestion. To account for lignocellulose-derived proteins, 71 corn proteins identified in the corn-stover samples in a preliminary analysis (data not shown) were added to the contaminants database. These proteins yielded 1,971 peptide spectrum matches (PSMs); however, 71.46% of them matched to tryptic peptides shared with *P. pulmonarius* proteins, making their origin ambiguous. To prevent confounding downstream quantification, these peptides were excluded from further analysis. Beechwood lacks an available proteome, and no matches were found using related *Fagaceae* proteomes from UniProt.

In addition to standard variable post-translational modifications (PTMs) such as methionine oxidation and protein N-terminal acetylation, we also included tryptophan oxidation, asparagine deamidation and pyroglutamate formation from N-terminal glutamine, based on an initial open search with FragPipe.^53,54^ Including these modifications, identified PSMs increased by 24.56% (Figure 2a). Despite the increase in PSM and peptidoform counts, the peptide count rose only by 4.45%, as most peptides were detected both with and without PTMs. Overall, we identified 17,201 unique peptides from *P. pulmonarius* proteins, yielding 24,307 distinct peptidoforms with the various PTMs.

**Figure 2:**
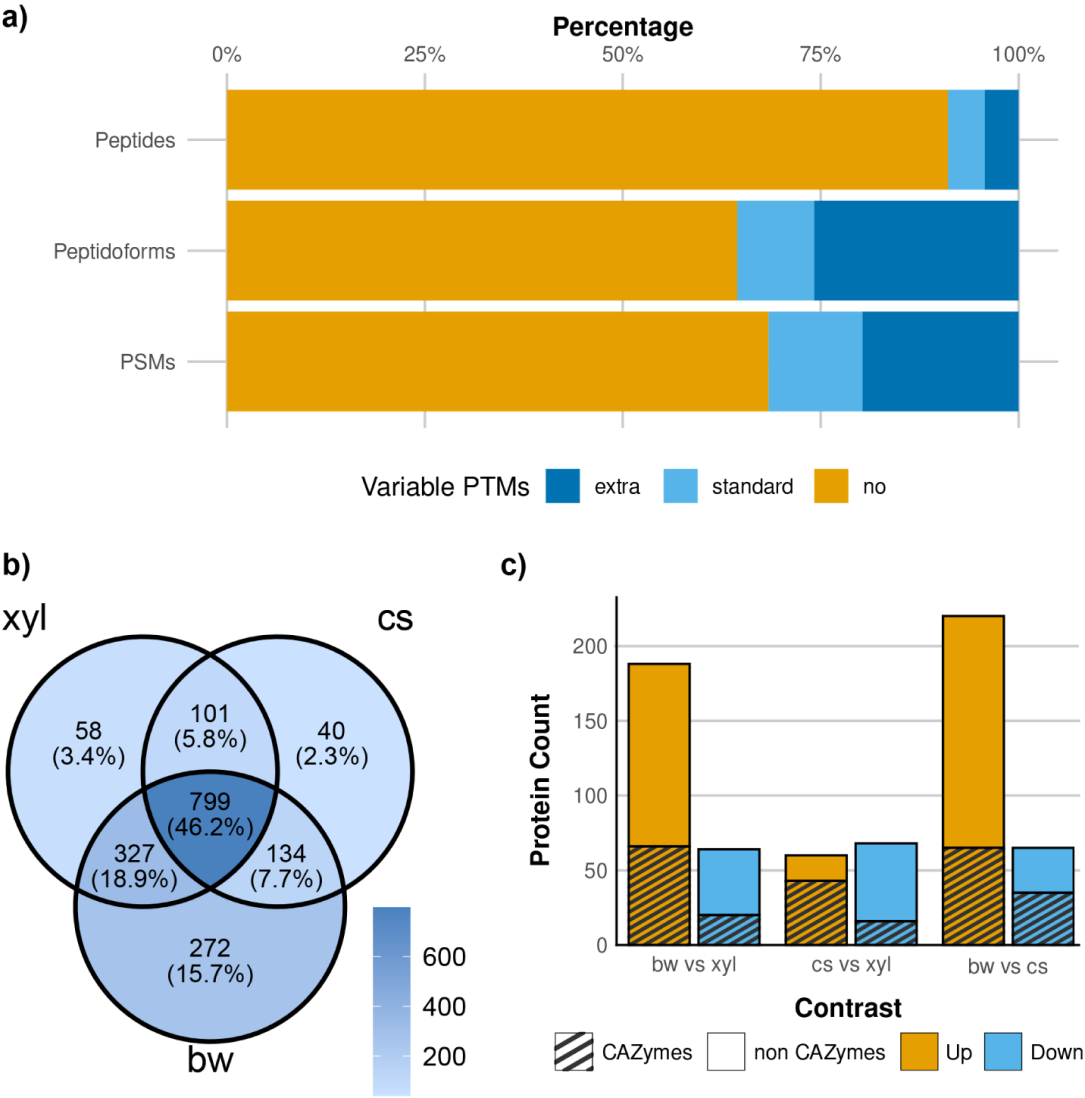
(a) Number of PSMs, peptidoforms, and peptides identified with no PTMs (yellow), standard PTMs (light blue), and extended PTMs (blue). (b) Overlap of quantified proteins across the three secretomes. (c) Number of differentially abundant CAZymes (striped), non CAZymes (nonstriped).

After removing contaminants and filtering for proteins detected in at least two replicates of any condition, 1,734 proteins were reliably quantified. Among them, 96.71% had at least two quantified peptidoforms, and 90.12% two unique peptides. Subcellular localization analysis predicted that 37.44% of quantified proteins were extracellular, while 11.84% were considered membrane-bound.

The secretome composition varied across substrates: only 799 proteins (46.16%) were shared among all conditions. A previous study on *P. ostreatus* reports an even smaller overlap of 20% secretome overlap between glucose and two lignocellulosic substrates.^15^ Beechwood induced the richest secretome, with 1,532 proteins, followed by xylose and corn stover (Figure 2b). Part of the higher number of beechwood identifications can be attributed to a technical factor: all beechwood replicates were run in a single batch on the same mass spectrometer, while corn stover and xylose spanned different batches and mass spectrometers and included runs that select fewer precursors for MS/MS, lowering the identification depth (see Text S1). Oxidoreductases, CAZymes and peptidases were the dominant protein classes as seen in other *Pleurotus* secretomes.^15,16,21^ Secreted peptidases contribute to fungal cell wall remodeling, recycling of extracellular organic nitrogen and enzyme activation.^67^ *P. pulmonarius* has been described as a peptidase-producing WRF, with a corn-derived substrate, corn bagasse, reported as the strongest inducer among different agro-industrial residues.^68^ However, our study showed a different trend. Beechwood triggered the highest abundance of peptidases, while corn stover yielded the lowest levels with most peptidases showing significantly reduced abundance in corn stover cultures compared to both beechwood and xylose (Table S2).

### CAZyme Secretion Profiles in Response to Lignocellulose

Beechwood induced the highest number of differentially abundant proteins compared to both xylose and corn stover. Interestingly, although more differentially abundant proteins were observed in xylose versus corn stover overall, this trend reversed at the CAZyme level: 43 CAZymes were significantly induced in corn stover relative to xylose, whereas only 17 showed the opposite pattern (Figure 2c). Additionally, CAZymes were more sensitive to substrate changes than non-CAZyme proteins: 43.34% of CAZymes were differentially abundant in at least one comparison, versus only 19.03% of non-CAZymes. This suggests a substrate-specific regulation for CAZyme secretion, distinct from broader protein expression patterns.

The genome of *P. pulmonarius* encodes 566 CAZymes, of which 56.9% were quantified in the secretomes. After excluding mostly intracellular glycosyltransferases (GTs), this proportion increases to 62.91%. Carbohydrate esterases (CE) had the highest (71.88%), and auxiliary activity (AA) enzymes the lowest representation in the secretomes (53.68%), a trend commonly observed in WRF.^7^ Carbohydrate-binding modules (CBMs) were also present: among 55 catalytic CAZymes with a CBM, 90.9% were detected.

### Coupling Biochemical and Proteomic Data

We coupled biochemical assays with proteomic data to interpret the enzymatic response of *P. pulmonarius* LGAM 28684 across substrates. From the combined secretomic and biochemical results (Figure 3) it was evident that corn stover secretomes showcased the highest enzyme activities for most hydrolases, while the beechwood secretomes were richest in laccase activity.

**Figure 3:**
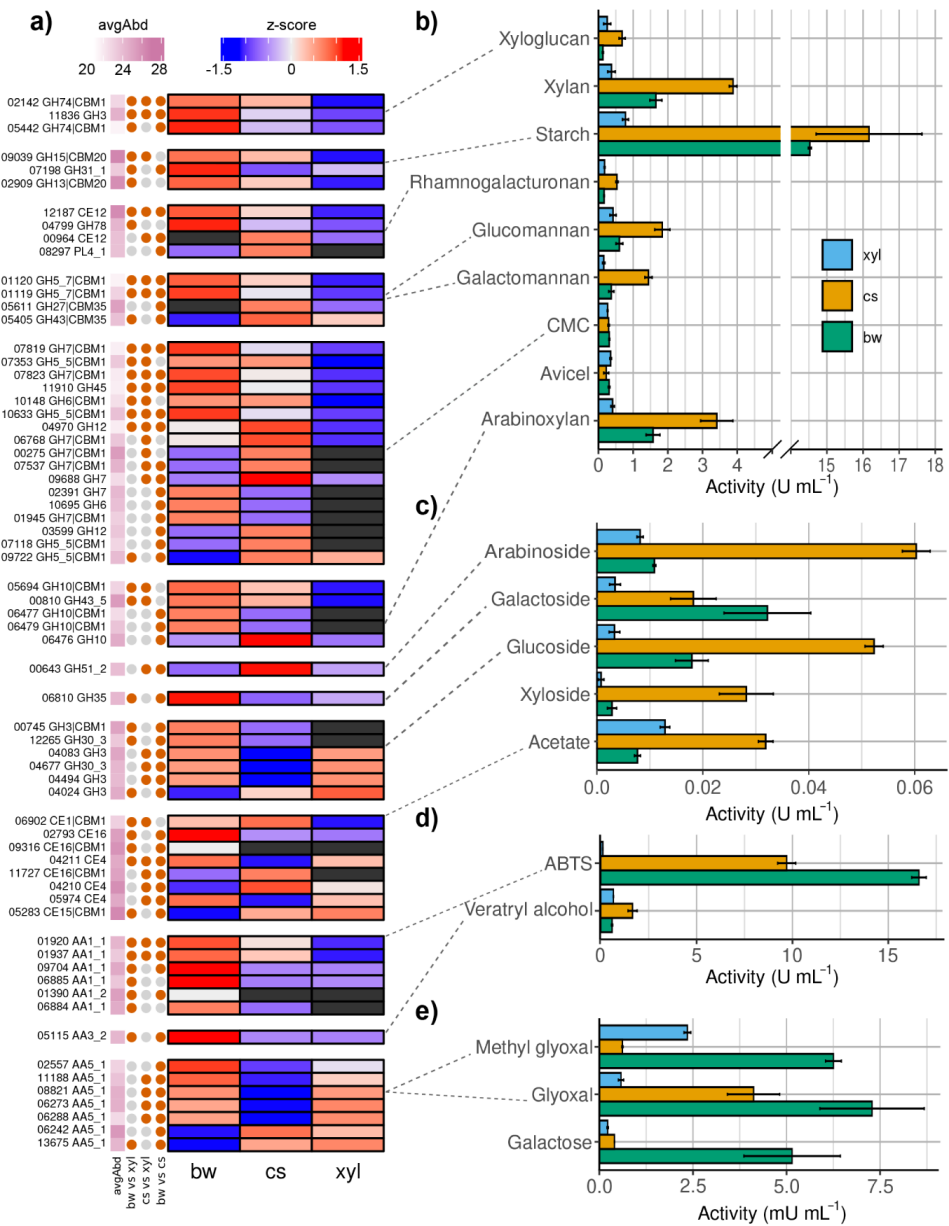
(a) Heatmap of z-scores of protein intensities for selected differentially abundant CAZymes; black color indicates not detected in the corresponding substrate. Protein accessions are shown without the AB1B83_0 prefix. Significant differential abundance in each comparison is marked with an orange circle. avgAbd: log_2_ of average protein intensity. Activity of the culture supernatants in (b) isolated polysaccharides, and (c) pNP- substrates (p-Np-α-D-arabinofuranoside, p-Np-β-D-galactoside, p-Np-β-d-glucopyranoside, o-Np-β-D-xyloside and p-NP-acetate). Oxidase activity of the culture supernatants for H_2_O_2_-independent (d) and H_2_O_2_-producing (e) oxidases.

#### Enzymes Acting on Isolated Polysaccharides

In our experiment, corn stover and beechwood were selected as representative substrates for arabinoxylan and glucuronoxylan, respectively. The highest glucuronoxylanase and arabinoxylanase activities were observed in corn stover cultures (Figure 3b). In CAZy, only two families, GH4 and GH115, contain enzymes reported to act specifically on glucuronoxylan. *P. pulmonarius* genome encodes a single GH115 α-1,2-glucuronidase (GenBank accession AB1B83_003742), but its expression remained stable across substrates. In contrast, multiple β-xylanases and α-L-arabinofuranosidases targeting arabinoxylan were detected in the secretomes. All four GH10 β-xylanases encoded in the genome were secreted; these xylanases tolerate xylan substitutions better, making them more effective on arabinoxylan than their GH11 counterparts.^69^ Two GH10 β-xylanases were overproduced in beechwood compared to corn stover and were absent in xylose, contrary to the peak of the relevant activity in the corn stover secretomes. Another GH10 xylanase (AB1B83_005694) was induced in both lignocellulosic substrates compared to xylose. The only β-xylanase lacking a CBM1 domain was induced in corn stover cultures relative to beechwood. Additionally, AB1B83_000810, an endo-α-1,5-L-arabinanase, was oversecreted in lignocellulose compared to xylose (Figure 3a).

High starch-degrading activity was detected after growth on both lignocellulosic substrates (Figure 3b). While corn stover may contain residual starch capable of inducing amylase activity, similarly high activity was observed in beechwood. This pattern may reflect a general strategy among *Pleurotus* species: *P. ostreatus* and *P. eryngii* predominantly secrete starch-degrading enzymes during early growth stages on lignocellulose.^67^

The activity measured toward microcrystalline and amorphous cellulose remained low (<1 U mL^–1^) (Figure 3d). Two other *Pleurotus* species, *P. ostreatus* and *P. eryngii*, are also reported to selectively degrade lignin and show very low activity on cellulose when grown on lignocellulosic material.^13,22^ Our proteomic analysis detected multiple cellulases, 48 out of the 64 cellulose-related genes encoded in the genome of *P. pulmonarius*. This included all three GH6 and all 12 GH7 genes, as well as five GH5_5|CBM1 genes. However, this substantial representation did not translate to high activity in biochemical assays. The lytic polysaccharide monooxygenases (LPMOs) related to cellulose degradation were also well represented. Of the 34 AA9 LPMO genes, 17 were present in the secretomes, but only two of them were found in xylose. The sole AA16 LPMO and the sole cellobiose dehydrogenase of the genome were also secreted only in the presence of lignocellulose (Table S2). These numbers reveal a potent oxidoreductive system for cellulose degradation, but this was not reflected in our biochemical data. Assays for LPMO activity did not reveal any oxidized sugars, since no peaks appeared in the chromatograms at around time 20–30 min.^58^ This indicates that the tested conditions were probably not optimal for LPMO activity (Figure S2).

Xyloglucan-degrading activity peaked in corn stover secretomes (Figure 3b), which was the only substrate that probably contains xyloglucan as an inducer. The genome encodes 11 relevant genes and six were quantified. Two enzymes acting on oligoxyloglucan were induced in lignocellulose, while the third (AB1B83_005442) was oversecreted in beechwood against both xylose and corn stover. The AB1B83_003599 GH12 was instead induced in corn stover compared to beechwood. As shown in our previous work,^56^ Basidiomycetes are fully equipped to tackle xyloglucan degradation, with multiple xyloglucanases that differ in substrate specificity and mode of action.

#### Accessory Enzymes

Esterases are critical for lignocellulose degradation, breaking the ester bonds linking hemicellulose to lignin, and thus contributing significantly in overcoming biomass recalcitrance. In the present study, pNP-acetate, a synthetic substrate that provides a general proxy for esterase activity, was used. Esterase activity was highest in corn stover cultures (Figure 3c), consistent with the substrate’s complex hemicellulose composition.^70^ Secretomic analysis revealed a rich diversity of carbohydrate esterases. All three CE1 genes were found in the secretomes and AB1B83_006902, a feruloyl esterase with a CBM1 module, was induced in the lignocellulosic substrates (Figure 3a). CE4 was the most abundant family, with 11 of 12 predicted genes detected. This included four acetyl xylan esterases of the CE4_e132 dbCAN-sub subfamily and seven GPI-anchored chitin deacetylases. These enzymes shared more than 75% sequence identity and identical lengths (248 amino acids), complicating their quantification. Among the quantified CE4, AB1B83_004211 and AB1B83_005974 were induced in beechwood but suppressed in corn stover, while AB1B83_004210 was strongly overproduced in corn stover compared to both other conditions (Figure 3a). CE16, the second largest esterase family in the genome, included three CBM1-containing enzymes detected in the secretomes, but out of the 8 CE16 without a CBM, only one was detected. One was overproduced in corn stover (AB1B83_011727), and two others in beechwood. The only glucuronoyl esterase (CE15; AB1B83_005283) in the genome was also detected and was less abundant in beechwood (Figure 3a).

In addition to esterase activity, several oligosaccharide-degrading enzymes were assayed using pNP substrates, including α-arabinofuranosidases, β-galactosidases, β-glucosidases and β-xylosidases. The activities measured for these enzymes remained low, consistently with their accessory action (Figure 3c). Enzymes like β-glucosidases and β-xylosidases are often found as membrane-associated enzymes, so their extracellular presence can be rather occasional and dependent on the experimental conditions.^71,72^ Nonetheless, the secretomes contained three α-L-arabinofuranosidases; two were stably expressed across conditions, while AB1B83_000643 was more abundant in corn stover (Figure 3a), in agreement with the high activity measured (Figure 3c). Two GH35 β-galactosidases were quantified in the secretomes. Among them, AB1B83_006810 was overproduced in beechwood, which was reflected in biochemical assays as well. All four β-xylosidases encoded in the genome, belonging to families GH3 and GH43, were detected in the secretomes. Although none showed differential abundance (Table S2), biochemical assays revealed higher β-xylosidase activity in corn stover supernatants. For β-glucosidases, divergence between biochemical and proteomic data was observed. We quantified 12 β-glucosidases spanning GH1, GH3, GH5_9, and GH30 families after filtering for EC numbers 3.2.1.21 and 3.2.1.75. Although the relevant activity peaked in corn stover supernatants, most β-glucosidases were induced in beechwood and xylose based on proteomics. This discrepancy could be attributed to the side activities of other β-glycosidases, which are abundant in the corn stover secretome. These enzymes could potentially hydrolyze pNP-β-glucoside as a side activity, leading to an overall increased β-glucosidase activity in this substrate. However, the fact that the abundance of β-glucosidases in beechwood was not reflected in the biochemical assays may also reflect a combination of technical and biochemical factors. Technical factors include the single-batch analysis of beechwood replicates increasing identification and quantification consistency compared to the other conditions (see Text S1). Additionally, bottom-up proteomics detects tryptic peptides of certain lengths, achieving limited sequence coverage and can miss features that can yield inactive proteoforms, such as truncations or certain PTMs.^73^ Biochemical factors include possible enzyme inactivation in the assay conditions due to the presence of inhibitors in the secretomes.

#### Oxidative Enzymes

Lignin is a major component of plant biomass, and Basidiomycetes are known for assimilating this recalcitrant polyphenol.^74^ In our study, *P. pulmonarius* secreted nine AA1_1 laccases and one AA1_2 ferroxidase, consistent with the high laccase activity measured in the assays (Figure 3d). Laccase activity was highest in beechwood cultures, followed by corn stover, and was minimal in xylose cultures where lignin was absent. Secretomic data mirrored these patterns: six and three laccases were overproduced in beechwood and corn stover relative to xylose, respectively (Figure 3a). These results are in line with our previous findings, highlighting the diversity of laccases produced by this strain.^26^

Unexpectedly, ligninolytic peroxidase activity was undetectable in biochemical assays, despite the secretomic identification of multiple peroxidases. No activity was observed with any of the tested H_2_O_2_-dependent assays, employing different substrates, including ABTS, DMAB-MBTH, Reactive Black 5, and veratryl alcohol. This is notable, given that *Pleurotus* species are among the few fungal genera known to produce versatile peroxidases (VPs).^6^ Five AA2 peroxidases, including two VPs, were quantified in the secretomes, at low levels, with none of them displaying differential abundance. The secretomes contained also two dye-decolorizing peroxidases (DyP), a family of ligninolytic peroxidases with low sequence similarity to the AA2 CAZy family.^75^ It is possible that the assay conditions did not support the activity or stability of these enzymes. Also, this could be due to the time frame of our experiment: for example, ligninolytic peroxidases in the *P. eryngii* wheat straw cultures were identified after day 14^16^.

Conversely, significant activity was detected for H_2_O_2_-producing oxidases against veratryl alcohol, galactose, and (methyl-) glyoxal substrates. H_2_O_2_-independent veratryl alcohol oxidation likely reflects aryl alcohol oxidase (AAO) activity (EC 1.1.3.7), which was particularly high and induced in corn stover (Figure 3d). The genome of *P. pulmonarius* encodes three AAOs of the AA3_e64 dbCAN_sub family, two of which are secreted. Interestingly, there are 18 more AA3_2 enzymes in the secretomes classified to five distinct dbCAN-sub subfamilies without substrate assignment. Two of them, AB1B83_007092 and AB1B83_004144, are induced in corn stover and could explain the activity peak. While *Pleurotus* species are known AAO producers,^76^ the diversity of uncharacterized AA3 enzymes observed here merits further investigation.

Glyoxal oxidase activity was induced in the presence of lignocellulose, peaking in beechwood supernatants (Figure 3e). In CAZy, glyoxal- and methyl glyoxal oxidases (EC 1.2.3.15) are classified within the AA5_1 subfamily, while galactose oxidases (EC 1.1.3.9) belong to AA5_2. *P. pulmonarius* encodes 16 enzymes of the AA5 family, all of which fall into the AA5_1 subfamily. The AA5_2 subfamily is largely restricted to *Ascomycota* and is absent from the Agaricomycetes class based on CAZy and Mycocosm data with *Pleurotus* being no exception. Nonetheless, galactose oxidase activity was detected (Figure 3e). This could be a side activity of AA5_1 enzymes, given their close relationship to AA5_2, but it could also be due to AA7 glucooligosaccharide oxidases, which are able to oxidize galactose with low affinity.^77^ Galactose oxidase activity was highest in beechwood cultures, probably due to AB1B83_002557 oxidase (Figure 3a). Both AA3 and AA5 oxidases are important accessory enzymes in lignocellulolytic systems, feeding H_2_O_2_ to peroxidases and providing a link between polysaccharide and lignin assimilation.

### Saccharification of Lignocellulosic Substrates

The complex enzymatic machinery characterized in this study, combining secretomic and biochemical data, proved highly effective in the saccharification of pretreated lignocellulose (Figure 4). Supernatants were added at the same protein loading across conditions.

**Figure 4:**
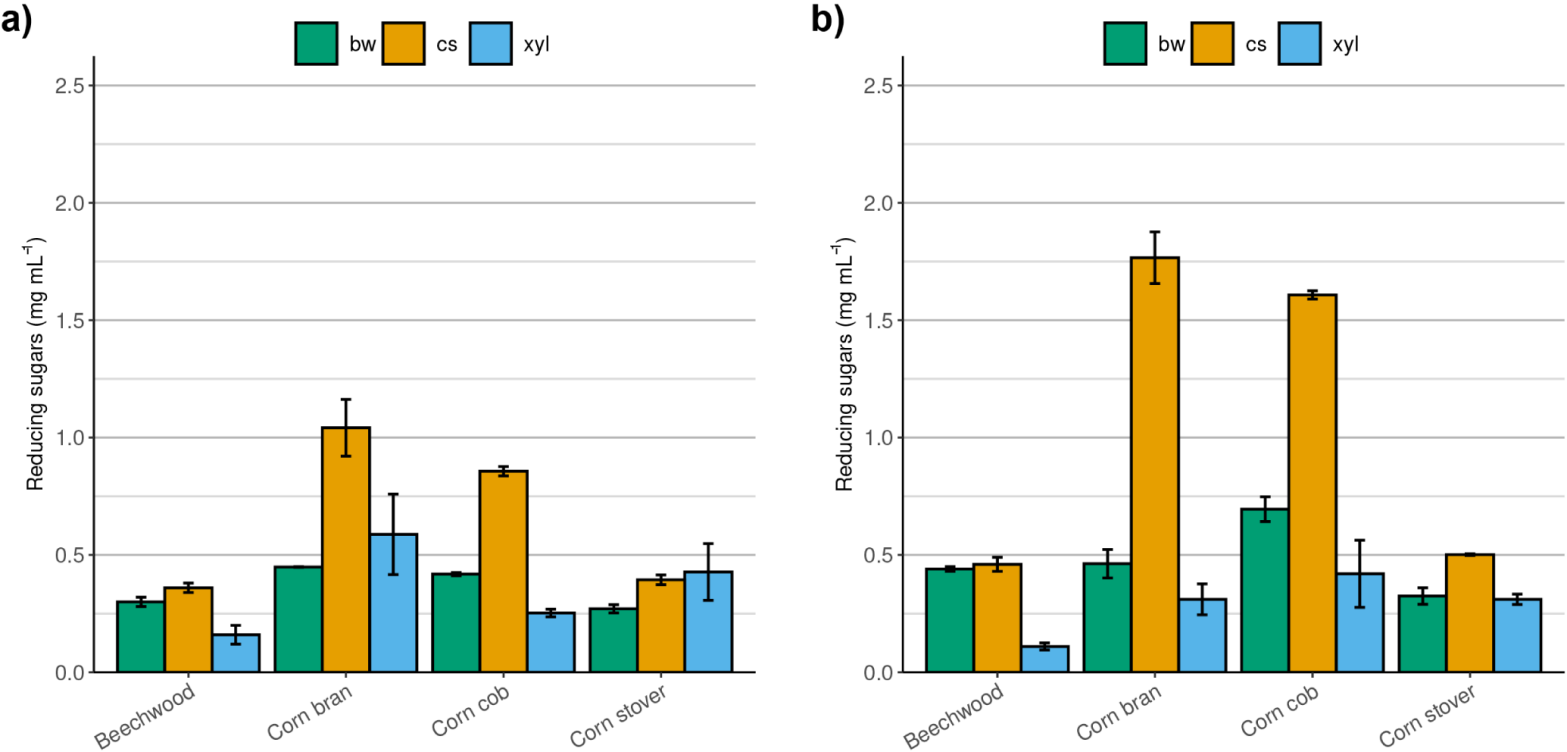
Saccharification of pretreated lignocellulosic substrates by the *P. pulmonarius* supernatants, without (a) and with (b) addition of NaN_3_. The protein loading was 5 mg protein mg^–1^ biomass.

In most cases, 0.5–1 mg mL^–1^ of reducing sugars were released from 50 mg mL^–1^ biomass. Supernatants from corn stover cultures were the most potent, releasing the highest sugar titer from two of the tested substrates. This is consistent with the notion that corn substrates induce the secretion of enzymes needed for their degradation.^78^ The same was not true for beechwood: the corresponding supernatants were less efficient in saccharifying pretreated beechwood compared to the corn stover secretome.

These results are consistent with our biochemical data, since higher polysaccharide-degrading activities were recorded in corn stover secretomes, in general (Figure 3b). Additionally, the heavily substituted corn xylan, worked probably as an inducer of enzymes acting on polysaccharide substituents with higher relevant activities in corn stover cultures (Figure 3c).

While broad enzyme diversity is often considered advantageous, only a fraction of the secretome may contribute effectively to biomass saccharification. Studies show that the deletion of noncontributive enzymes can improve the secretion of other enzymes and the overall activity of the mixture.^79^ Corn stover secretomes had less induced proteins than beechwood, but a very high proportion of CAZymes (Figure 2C). Thus, the more targeted enzyme profile of corn stover secretome, with less redundancy and higher proportion of effective enzymes, may contribute to their superior saccharification performance.

A caveat in our analysis and interpretation is the identification of multiple CAZy subfamilies without substrate prediction and of uncharacterized proteins without known functional domains and motifs. These proteins might also contribute to lignocellulose degradation in unknown ways. The analyzed secretomes contain 82 secreted uncharacterized proteins, 53 of which are classified as small secreted proteins (SSPs).^80^ A studied family of SSPs associated with lignocellulose degradation in *P. ostreatus* is encoded by the ssp1–6 genes, with transcriptional upregulation of ssp6 associated with reduced lignin degradation.^81^ In our experiment, only the ortholog of ssp6, AB1B83_002513, was quantified and was less abundant in beechwood, compared to both xylose and corn stover. However, for the vast majority of the detected SSPs there is promising potential to yield yet undiscovered functions.^80^

#### Oxidoreductase Deactivation Increases Reduced Sugar Release

Interestingly, as shown in Figure 4b, the inhibition of oxidoreductases might apparently increase biomass saccharification *in vitro*. When 0.2% (w/v) NaN_3_ was added to the reactions, reducing sugar yields from corn stover supernatants nearly doubled, reaching 1.76 ± 0.11 mg mL^–1^ for corn bran and 1.61 ± 0.02 mg mL^–1^ for corn cob. Beechwood supernatants also performed better after the addition of NaN_3_, although the effect was more pronounced for corn stover. This improvement suggests that oxidoreductases may inhibit polysaccharide-degrading enzymes during *in vitro* saccharification. Low molecular weight phenolics, produced by laccase-mediated oxidation of lignin, can inhibit β-glucosidases and xylosidases.^82^ This contrasts with the *in vivo* strategy of WRF for biomass degradation, which rely heavily on oxidoreductases.^8^ A plausible explanation is that lignin degradation products are assimilated and readily metabolized from the fungus in nature,^74^ and therefore, removed from the vicinity of glycoside hydrolases. On the contrary, when only the extracellular enzyme extract is used, lignin degradation products remain in the reaction medium, acting as inhibitors for hydrolases. Another possibility worth considering is that WRF may employ oligosaccharide oxidases as part of their oxidoreductive degradation mechanisms. Five AA7 enzymes were present in *P. pulmonarius* secretomes (Table S1), which are known to oxidize the reducing ends of various oligosaccharides.^83^ It is unclear whether NaN_3_ inhibits these oxidases. If it does, the observed increase in measured sugars might reflect a rise in unmodified reducing ends, rather than an overall sugar concentration increase. The importance of these enzymes in native WRF secretomes remains unknown, as they were recently discovered and the literature is limited.^77^ In contrast, NaN_3_ does not inhibit LPMO activity.^58,84^ In order to test this hypothesis, we performed reactions with the *P. pulmonarius* corn stover secretomes in the absence of NaN_3_, and the reaction supernatants, after enzyme thermal deactivation, were transferred to fresh substrate and enzyme with NaN_3_, and incubated further. If laccase-produced phenolics were responsible for hydrolases inhibition, the inhibitors present in the reaction supernatant should still inhibit hydrolytic enzymes, despite the addition of NaN_3_ the second day of the reaction. This was not the case, since reducing sugar production the second day of the reaction was almost 3-fold (1.8 ± 0.12 mg mL^–1^ and 4.5 ± 0.36 mg mL^–1^ the first and second day, respectively). This result indicates that glycooligosaccharide oxidases may have a more impactful role in the lignocellulose-degrading strategy of WRF, than previously thought, and they can hinder correct estimation of the hydrolysis by the 3,5-dinitrosalicylic acid (DNS) method. The increase in reducing sugars with the addition of NaN_3_, can have significant implications on the design of next-generation WRF-based enzyme cocktails for commercial applications, raising multiple questions regarding the exploitation of these systems in industrial settings. Also, the results presented in this work highlight a gap in our current understanding of the interplay between different CAZymes in biomass saccharification. Future work is needed to elucidate these phenomena *in vitro*, and determine the best strategies to develop industrially relevant WRF-based enzyme cocktails.

### Supplementation of a Cellulolytic Cocktail with *P. pulmonarius* Secretome for Corn Bran Saccharification

To evaluate the synergistic potential of *P. pulmonarius* enzymes with an industrial cellulase cocktail, we supplemented CellicⓇCTec2 with corn stover culture supernatants of*P. pulmonarius*. Saccharification was tested on pretreated corn bran, with a fixed total protein load of 5 mg g^–1^ biomass in all reactions (Figure 5).

**Figure 5:**
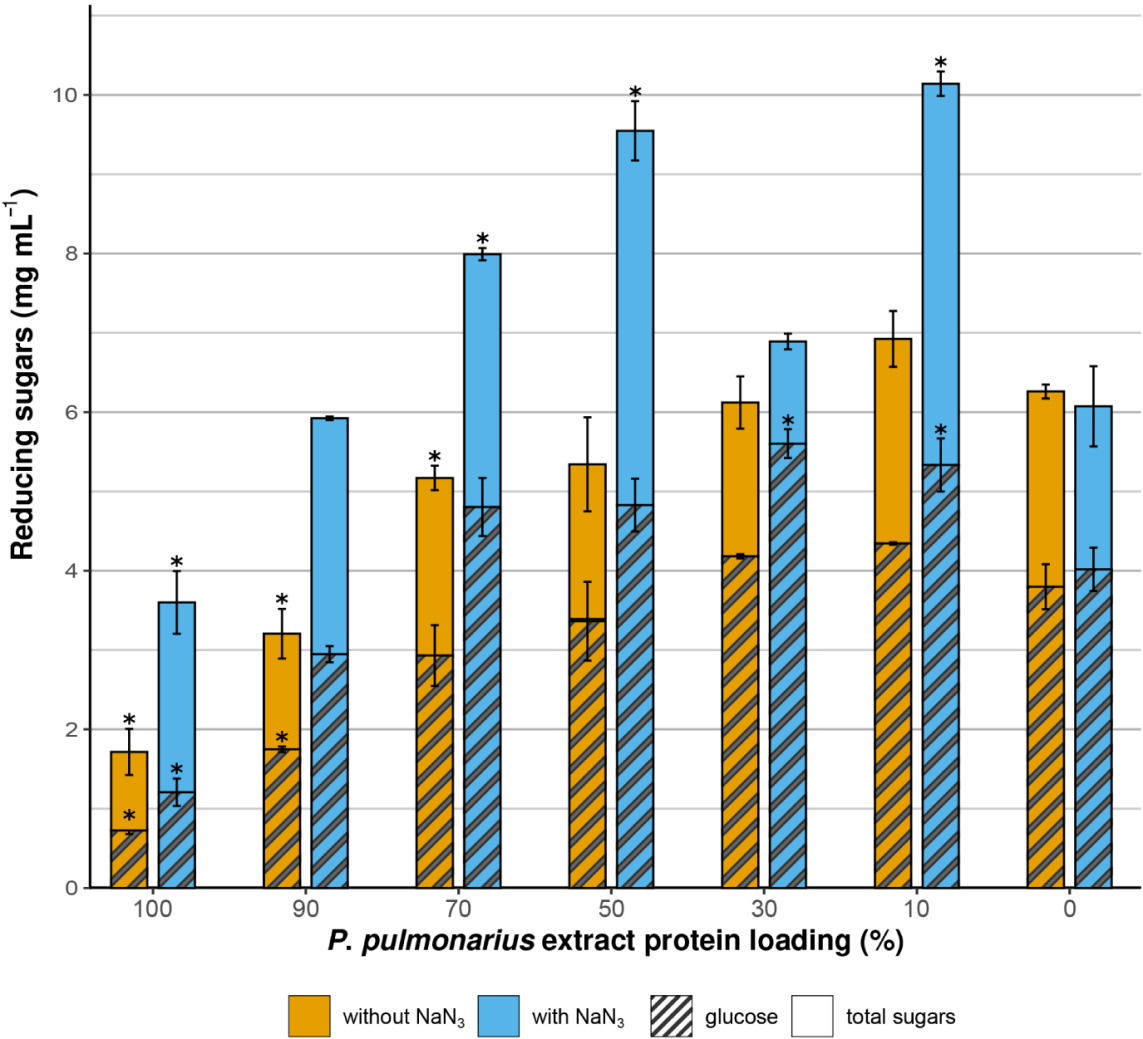
Saccharification of pretreated corn bran with the combined action of CellicⓇCTec2 and the *P. pulmonarius* culture supernatant, after growth in corn stover. The total protein content of the reaction was 5 mg g^–1^ biomass. Asterisks indicate significantly different data, compared to the respective “0” sample, which represents that the total protein added in the reaction corresponds to CellicⓇCTec2. Υellow: reactions without NaN_3_, light blue: reactions with 0.2% (w/v) NaN_3_, stripped: glucose concentration, nonstripped: total reducing sugars concentration.

Increasing proportion of the total protein content was substituted with *P. pulmonarius* extracts. Without NaN_3_, sugar release remained stable up to 50% *P. pulmonarius* extract but declined at higher levels. NaN_3_ had no effect on CellicⓇCTec2 performance alone, which is expected since the LPMOs present in the enzyme mixture are not affected by it. However, in contrast with WRF, *Trichoderma reesei*, the source of CellicⓇCTec2, is a potent producer of cellulose-degrading enzymes. This is not the case for the production of accessory oxidases, including laccases and AA7 glycooligosaccharide oxidases, from this organism^85^ suggesting that these oxidases play a minor role in this system. When NaN_3_ was added in the presence of *P. pulmonarius* culture supernatants to inhibit oxidoreductases, the synergistic enzyme system performed markedly better. Replacing just 10% of the total protein content with *P. pulmonarius* extract increased total reducing sugar release by 40% and glucose release by 33% compared to the control (100% CellicⓇCTec2). This enhanced saccharification is observed up to 70% of *P. pulmonarius* extract supplementation. Given the low glucose yield when the secretome was used alone, it is likely that most glucose is released from the action of CellicⓇCTec2 cellulases. These results indicate that *P. pulmonarius* enzymes enhance the performance of the cellulase cocktail and highlight their potential as additives in the design of efficient enzyme cocktails for biomass biorefinery applications.

### *P. pulmonarius* Is a Versatile Tool for the Production of Substrate-Specific Enzyme Mixtures

This study demonstrates that *P. pulmonarius* shows a highly dynamic enzymatic response when grown on different lignocellulosic substrates, producing a full complement of enzymes for the breakdown of plant biomass. While most WRF, including *Pleurotus*, are considered selective lignin degraders, our data reveal a notable polysaccharide-degrading capacity. The fungus secreted a broad array of both polysaccharide-degrading and accessory enzymes, together with lignin-acting oxidases, enabling the degradation of most biomass components. Moreover, each substrate induced a distinct secretome profile, supporting the potential of *P. pulmonarius* as a platform for customized enzymatic cocktails that complement existing industrial preparations. The versatility of the enzymatic response of the strain toward different substrates could be harnessed as a powerful tool toward the design of next-generation, WRF-based enzyme cocktails, tailored for specific feedstocks and applications. The relative lack of cellulase activity, at least in the tested conditions, could be exploited in applications where cellulose should be left intact, i.e., the production of nanocellulose. Overall, this study underscores the versatility of *P. pulmonarius* in adaptation to diverse agro-industrial residues and its value as a source of tailored enzymatic cocktails for future biorefineries.

## Supplementary Material





## Data Availability

This Whole Genome Shotgun project has been deposited at DDBJ/ENA/GenBank under the accession JBHEFG000000000. The MS proteomic data along with all metadata are deposited to the ProteomeXchange Consortium via the PRIDE^86^ partner repository with the data set identifier PXD065010 and 10.6019/PXD065010. All code for the analysis is available in GitHub (https://github.com/Roman-Si/Pleurotus_proteomics). Data from the Mycocosm, UniProt and GenBank databases were accessed in March 2025.

## Notes

Table S1: CAZyme annotations in the genome of *P. pulmonarius* LGAM 28684; Table S2: Results of the proteomic analysis (XLSX)
Text S1 contains extended material and methods; Figure S1: K-mer spectrum of raw DNA reads; Figure S2: HPAEC-PAD chromatograms of the *P. pulmonarius* culture supernatants’ reaction on PASC (PDF)

## Abbreviations

AA: auxiliary activity
AAO: aryl-alcohol oxidase
CAZy: carbohydrate-active enzyme
CBM: carbohydrate-binding module
CE: carbohydrate esterase
CMC: carboxymethylcellulose
DNS: 3,5-dinitrosalicylic acid
EC: enzyme commission
GH: glycoside hydrolase
HMM: Hidden Markov Model
LC: liquid chromatography
LCB: lignocellulosic biomass
LPMO: lytic polysaccharide monooxygenase
MS: mass spectrometry
NP: nitrophenyl
PASC: phosphoric acid swollen cellulose
PSM: peptide-spectrum match
PTM: post-translational modification
TEAB: triethylammonium bicarbonate
TMP: Tukey’s median polish
UFBoot: ultrafast bootstrap
VP: versatile peroxidase
WRF: white-rot fungi
