# Supplementary material for: Genomic, Proteomic, and Biochemical Study of Pleurotus pulmonarius Secretome and Its Role in Biomass Saccharification
Source: J Agric Food Chem. 2025 Oct 27;73(44):28539–51. doi: 10.1021/acs.jafc.5c09170 (PMC12593350; doi:10.1021/acs.jafc.5c09170)
Supplement: Supplementary file 2 [file jf5c09170_si_002.pdf]

# Genomic, proteomic and biochemical study of *Pleurotus pulmonarius* secretome and its role in biomass saccharification

Romanos Siaperas[1], Eftychia Papadaki[2], Panagiotis Giannikos[1], Anastasia Zerva[2*], Evangelos Topakas[1,3*]

[1]Industrial Biotechnology & Biocatalysis Group, Biotechnology Laboratory, School of Chemical Engineering, National Technical University of Athens, 9 Iroon Polytechniou Street, Athens, Attiki, 15772 Athens, Greece

[2]Laboratory of Enzyme Technology, Department of Biotechnology, School of Applied Biology and Biotechnology, Agricultural University of Athens, 75 Iera Odos Street, Athens, Attiki, 11855 Athens, Greece

[3]Biochemical and Chemical Process Engineering, Division of Sustainable Process Engineering, Department of Civil, Environmental and Natural Resources Engineering, Luleå University of Technology, Luleå, Sweden

[*]Correspondence: E. Topakas. Tel: +30-210-7723264; fax: +30-210-7723163; e-mail: vtopakas@chemeng.ntua.gr, A. Zerva Tel: +30210529 4316; e-mail: anazer@aua.gr.

# Text S1

## Materials, enzymes, substrates

Chemicals were purchased from Applichem (Darmstadt, Germany) or Sigma-Aldrich (St. Louis, MO, USA) and were of the highest available purity. The commercial cellulolytic enzyme preparation Cellic®Ctec2 (Batch no: VCNI0001) was kindly gifted by Novozymes A/S (Bagsvaerd, Denmark). Carboxymethylcellulose (CMC), starch, and microcrystalline cellulose (Avicel) were supplied by Sigma-Aldrich. All other polysaccharides (tamarind xyloglucan, rhamnogalacturonan, carob

galactomannan, glucomannan, rye arabinoxylan, wheat arabinoxylan, beechwood xylan), along with pNP-α-L-arabinofuranoside were purchased from Megazyme (Bray, Co. Wicklow, Ireland). All other nitrophenyl-derived substrates (pNP-β-D-glucopyranoside, pNP-β-D-galactopyranoside, o-nitrophenyl-β-D-xylopyranoside, and pNP-acetate) were obtained from Alfa Aesar (Ward Hill, Massachusetts, USA) or Sigma-Aldrich. All other chemicals were purchased from Merck KGaA (Darmstadt, Germany) and were also of the highest purity available.

Lignocellulosic materials (corn bran, corn cob, and corn stover) were pretreated as described in Bakouli et al.[1]. Beechwood (Lignocel HBS 150-500) was hydrothermally pretreated as described in Katsimpouras et al.[2].

## Structural and function annotation

Gene prediction was carried out with BRAKER v3.0.3[3] using the fungal partition of orthoDB v11 and RNAseq reads from *P. pulmonarius* CCMSSC04423 (SRA accessions: SRR6370795, SRR6370796, SRR6370792). Gene and isoform models were reduced using TSEBRA[4] and supplemented into MAKER v2.31.11[5] along with the BRAKER-assembled transcripts to append untranslated regions. Gene models lacking transcript support and without InterPro annotations or BLAST hits in the fungal partition of orthoDB v11 (>= 70% coverage, >= 30% identity) were discarded.

Gene and product names were assigned using BLAST against SwissProt after filtering the alignments with Haas et al.[6] criteria, UniFIRE v2024_02, or InterProScan v5.66-98.0 filtered at e-value <1e-15 and protein coverage > 70%. CAZymes were identified using run_dbcan v4.1.4 with DIAMOND against the CAZy FASTA (release 07142024) and HMMER3 against dbCAN HMMs v13 and dbCAN_sub[7]. HMMER3 hits were filtered with an e-value of 1e-17 and coverage of 45%. DIAMOND-only hits were filtered at 95% query and subject coverage and manually cross-validated with InterProScan and UniFIRE annotations.

## Phylogenetic analysis

*P*. *pulmonarius* LGAM 28684 was previously misidentified as *P*. *citrinopileatus*. To resolve its taxonomy, a phylogenetic analysis of the *Pleurotus* genus was performed using data from a phylogenetic analysis of the *P*. *ostreatus* species complex based on 40 nuclear single-copy orthologous genes. Gene sequences for 49 *Pleurotus* isolates and two *Hohenbuehelia* outgroups of Li et al.[8] were retrieved from GenBank.

Next, we appended the *Pleurotus* annotated genomes to this dataset. The corresponding orthologous regions were identified using BLAST with the Li et al. sequences as templates, retaining hits with >= 70% identity and query coverage, and ensuring that the top-scoring templates aligned in the same region. The genome of *P*. *ostreatoroseus* (accession GCA_005298045.1) was excluded as it contained only three of the 40 gene markers.
Orthologs were aligned with MAFFT v7.490 and trimmed with ClipKIT v2.3.0[9,10]. Maximum-likelihood phylogenetic analysis was conducted with IQ-TREE v2.3.6 with an edge-linked, fully-partitioned model[11]. Node support was assessed via 1,000 ultrafast bootstrap (UFBoot) replicates. Tree parsing and visualization were performed with the ggtree Bioconductor package[12].

## Cultivation of *P. pulmonarius* for secretome analysis

Fungal biomass was first grown in 100 mL precultures with xylose (5.7% w/v) as the sole carbon source for 10 days. The medium also contained 30 g $L^{-1}$ yeast extract, 1 g $L^{-1}$ $K_2HPO_4$, 0.2 g $L^{-1}$ $MgSO_4 \cdot 7H_2O$, and the pH was adjusted to 6 with HCl[13]. Mycelia were then aseptically washed and transferred to fresh medium containing either corn stover or beechwood (4% w/v) as carbon source. In order to avoid the interference of yeast extract peptides in the proteomic analysis, the nitrogen source in the main cultures was replaced with 1 g $L^{-1}$ $NH_4NO_3$ and 2 g $L^{-1}$ $(NH_4)_2C_4H_4O_6$. Xylose-grown cultures with the same nitrogen source were freshly inoculated and maintained in parallel as controls[13]. After 10 days of growth at 27 °C and 100 rpm, culture supernatants were isolated by filtration and concentrated 10-fold using a 10 kDa cutoff polyethersulfone membrane in an Amicon ultrafiltration device. For enzymatic assays and synergism experiments, the concentrated

supernatant was used directly. For proteomic analysis, supernatants were concentrated, freeze-dried, and stored at -20 °C. Protein concentration was determined using the Bradford assay.

## Proteomic analysis

Samples included three replicates for each condition and were processed in two batches at the VIB Proteomics Core (Ghent, Belgium) for label-free LC-MS/MS analysis. The first batch included one replicate of corn stover and two replicates of xylose. The remaining samples were in the second batch.

### Sample preparation for proteomics

Sample preparation was identical for both batches. Protein extracts were dissolved in 5% sodium dodecyl sulfate (SDS) in 50 mM triethyl ammonium bicarbonate (TEAB) to a final concentration of 1 µg $µl^{-1}$. Retained proteins were reconstituted in 300 µl of 5% SDS in 50 mM TEAB, reduced with 15 mM dithiothreitol (DTT) at 55 °C for 30 minutes, and alkylated with 30 mM iodoacetamide at room temperature in the dark for 15 minutes. Phosphoric acid was added to a final concentration of 1.2% to lower the pH to ~1, followed by the addition of 7 volumes of 90% methanol in 100 mM TEAB (pH 7.55). Samples were loaded onto mini S-trap columns (Protifi) in 400 µl aliquots, centrifuged at 4000 x g for 30 seconds, and washed three times with 400 µl of 90% methanol TEAB. Proteins were digested on-column overnight at 37 °C with 1 µg MS-grade trypsin (Promega) in 125 µl of 50 mM TEAB. Peptides were eluted in three consecutive centrifugations (1 min at 4000 x g each) using 80 µL of 50 mM TEAB, 80 µL of 0.2% formic acid, and 80 µL of 50% acetonitrile. Eluted peptides were transferred to MS vials and dried completely.

### Peptide separation

Dried peptides were dissolved in 20 µL of loading solvent A (0.1% trifluoroacetic acid (TFA) in water/acetonitrile, 98:2 v/v).

**Batch 1**: 3 µL (5 µL for PRC-5590) were loaded onto an in-house trapping column (100 µm internal diameter (I.D.) × 20 mm, 5 µm beads Reprosil-HD C18, Dr. Maisch, Germany). Separation was achieved on a 50 cm µPAC™ column using a stepped gradient from 98% solvent A′ (0.1% formic acid in water) to 30% MS solvent B′ (0.1% formic acid in 80% acetonitrile) over 135 minutes, to 50% B′ in 15 min, followed by a 5-minute wash up to 95% B′ (70% for PRC-5590). The flow rate was 250 nL $min^{-1}$.

**Batch 2**: 10 µL were injected onto an Ultimate 3000 RSLCnano system (Thermo Scientific). Trapping was performed at 20 µl $min^{-1}$ for 2 min on a 5 mm C18 trapping column (300 µm I.D., 5 µm beads, Thermo Scientific). Separation was carried out on a 250 mm Aurora Ultimate column (1.7µm C18, 75 µm I.D., Ionopticks) at 45 °C using a non-linear gradient from 0.5% to 26% MS solvent B over 135 minutes, reaching 44% at 155 minutes, followed by a 10-minute wash at 56% and re-equilibration with 0.1% formic acid in water. Flow rate was 300 nL $min^{-1}$.

## MS/MS data acquisition

**Batch 1**: Samples were analyzed on a Q Exactive mass spectrometer (Thermo Scientific) operated in positive ion data-dependent acquisition (DDA) mode. MS1 scans were acquired over 400-2000 m/z at 70,000 resolution (200 m/z), with an AGC target of $3\times10^6$ and maximum injection time of 80 ms. The top 5 most intense ions with charges 2-4 were selected for MS2 (resolution 17,500) with predefined selection criteria: AGC target of 50.000, isolation window of 2 Da, fixed first mass of 140 m/z, centroid mode, intensity threshold of $1.3e^4$, exclusion of isotopes and dynamic exclusion set to 12 s. Fragmentation used HCD at 25% normalized collision energy. The ion 445.120025 Da (polydimethylcyclosiloxane) was used as internal lock mass.

**Batch 2**: Samples were analyzed on a Q Exactive HF Biopharma mass spectrometer (Thermo Scientific). MS1 scans were acquired over 375-1500 m/z at 60,000 resolution, with an AGC target of $3x10^8$. The 12 most intense ions (intensity >15,000) were isolated with a width of 1.5 m/z for fragmentation with HCD at 28% normalized collision energy. MS2 scans (200-2000 m/z) were

acquired at 15,000 resolution in the Orbitrap after accumulation of 100,000 ions for up to 120 ms. The internal lock mass ion was 445.120028 Da.

**Data processing**

Raw files were searched together using the nf-core/quantms v1.3.1dev pipeline of the nf-core collection of workflows against the proteome of *P. pulmonarius* LGAM 28684 and a contaminant database with common proteomics contaminants and 71 corn proteins identified in a preliminary search against the corn proteome (UniProt accession: UP000007305). Mass tolerances were defined based on Param-Medic[14]: 40 ppm for precursor and 0.02 Da for fragment ions. Trypsin cleavage was allowed a maximum of two missed cleavages. Searches were performed with Comet, MS-GF+ and SAGE, with rescoring by MS2Rescore. Variable modifications included methionine and tryptophan oxidation, protein N-termini acetylation, asparagine deamidation, and pyroglutamic acid formation from N-terminal glutamine. Carbamidomethylation of cysteine residues was set as a fixed modification. Variable modifications were selected based on a preliminary open search using Fragpipe with default parameters[15,16].

Peptide intensities were imported into the prolfqua R package, $\log_2$ transformed, and robust z-score scaled. Protein intensities were estimated using Tukey's median polish (TMP). However, in cases where all peptides of a protein were unique to single samples, TMP produced artificial uniform intensity values across all samples (as previously noted in microarray analyses[17]). In these cases, the median of the top three peptides was used instead. Proteins quantified in at least two biological replicates of any condition were retained for downstream analysis. Differential protein abundance was tested using the empirical Bayes approach with imputation of missing values as implemented in prolfqua. The experimental and MS/MS batches were combined and included as a covariate in the linear model following recommendations of Nygaard et al.[18].

## Enzyme activities and synergism assays

**CAZymes activity on isolated polysaccharides and model substrates**

Enzyme assays were performed with 0.5% (w/v) of each polysaccharide (konjac glucomannan, carob galactomannan, microcrystalline cellulose, CMC, beechwood xylan, tamarind xyloglucan, wheat arabinoxylan, rhamnogalacturonan and starch) in 100 µL reactions in 100 mM phosphate-citrate buffer pH 5. Reactions were incubated for 30 min at 40 °C and 1200 rpm in an Eppendorf thermomixer. Reducing sugars were quantified by the DNS method after stopping the reactions with 100 µL DNS reagent. Assays with nitrophenyl (NP)-derived substrates (pNP-β-D-glucopyranoside, pNP-β-D-galactopyranoside, oNP-β-D-xylopyranoside, and pNP-acetate) were performed 200 µL final volume, with substrates at 2 mM in 100 mM phosphate-citrate buffer (pH 5). After 15 min incubation at 40 °C, reactions were stopped with 40 µL $Na_2CO_3$ 1M, and absorbance was read at 410 nm. Product quantification was performed using suitable calibration curves.

Laccase activity was measured using ABTS as substrate[19]. Aryl alcohol oxidase activity was measured with 2 mM veratryl alcohol in 100 mM phosphate-citrate buffer pH 5. Product formation was monitored at 310 nm ($\varepsilon_{310}$=9300 $M^{-1}$ $cm^{-1}$) at 40 °C for 30 min. Galactose and glyoxal oxidase activities were assayed with an HRP-phenol red coupled assay, as previously described[13], using 1 mM galactose, glyoxal or methyl glyoxal as substrates. LPMO activity was assessed using 0.5% (w/w) phosphoric acid swollen cellulose (PASC) and 1 mM cysteine as reductant. The supernatants were analyzed for oxidized products with HPAEC-PAD[20]. Appropriate blanks were used in all cases, without the addition of enzyme. 1 Unit of enzyme is defined as the amount of the enzyme releasing 1 µmol of product per min at the above conditions.

**Activity on lignocellulosic biomass**

Enzyme extracts were tested on pretreated lignocellulose biomass (corn stover, corn bran, corn cob or beechwood) in 500 µL reactions containing 50 mg $mL^{-1}$ biomass and 100 mM phosphate-citrate buffer pH 5, with or without 0.2% (w/v) sodium azide. The protein loading was 5 mg $g^{-1}$ of biomass. Reactions were incubated at 40 °C and 1200 rpm for 24 h in an Eppendorf Thermomixer. Reducing

sugars were quantified in the supernatant using the DNS method[21]. All reactions were accompanied with appropriate blanks, where thermally inactivated secretome was added, with or without the addition of sodium azide, in order to correct for background absorbance.

### Synergism assays with commercial cellulases

Synergism assays were performed using the commercial cellulase preparation Cellic®CTec2. Total protein loading was set to 5 mg $g^{-1}$ of biomass. The biomass used as substrate was pretreated corn bran at a final concentration of 50 mg $mL^{-1}$. Reactions were performed at a final volume of 0.5 mL, with or without 0.2% (w/v) sodium azide. For each condition, a fraction of the total protein load was replaced by *P. pulmonarius* secretome extract obtained from corn stover cultures. Reactions were incubated at 40 °C and 1200 rpm for 24 h in an Eppendorf thermomixer. Then, the mixtures were centrifuged, and the supernatants were boiled to stop the reaction. Reducing sugars were quantified using the DNS method[21]. Glucose was quantified using the glucose oxidase method (GO kit, Merck). All reactions were accompanied with appropriate blanks, where no enzyme was added, with or without the addition of sodium azide, in order to correct for background absorbance. For the calculation of the reducing sugars released by the enzyme action, the inherent sugar content of the crude enzyme preparations (both *P. pulmonarius* secretome and Cellic®Ctec2) was subtracted by the total reducing sugars measured by the DNS method, after calculating the respective dilutions.

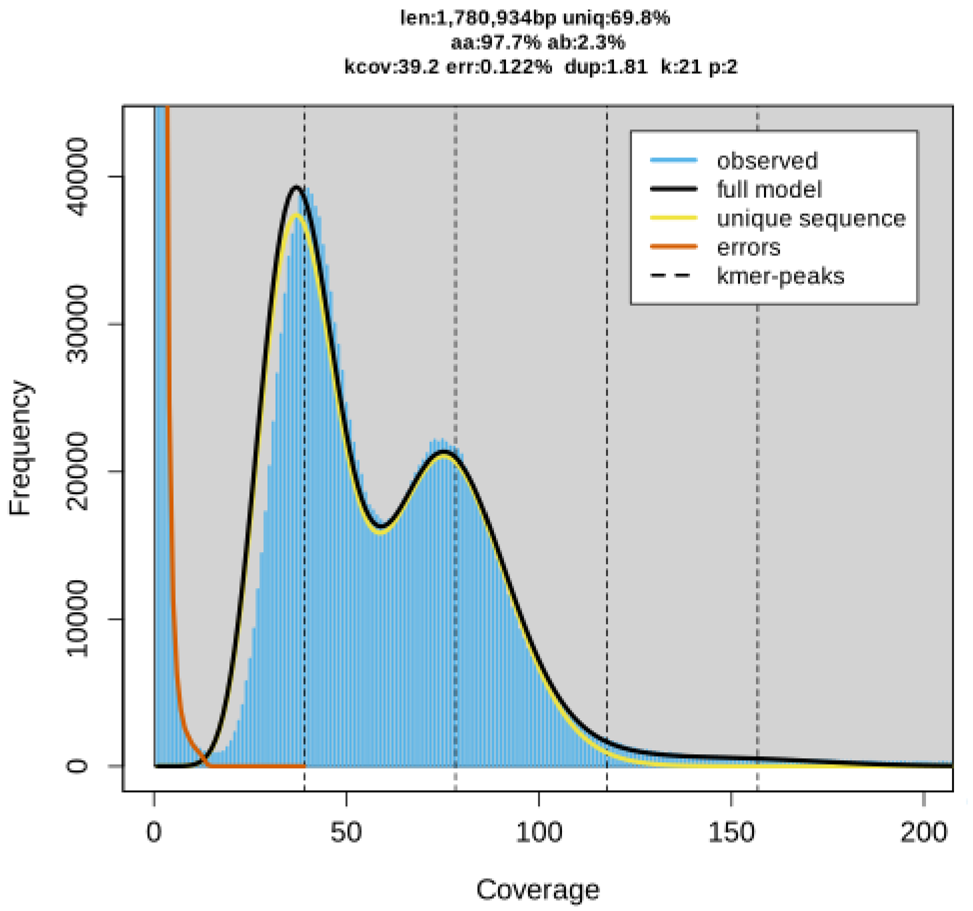


**Figure S1:** *K-mer spectrum of raw DNA reads. K-mer length was 21.*

(a)

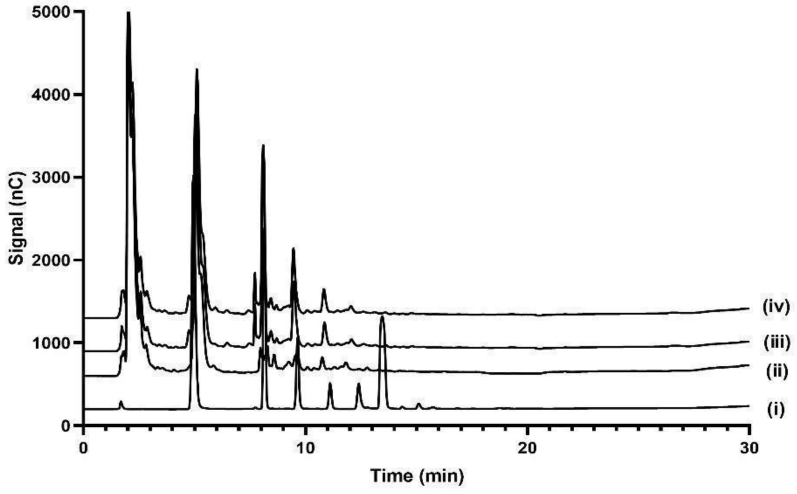

5000
4000
3000
2000
1000
0
Signal (nC)
0
10
20
30
Time (min)
(iv)
(iii)
(ii)
(i)


(b)

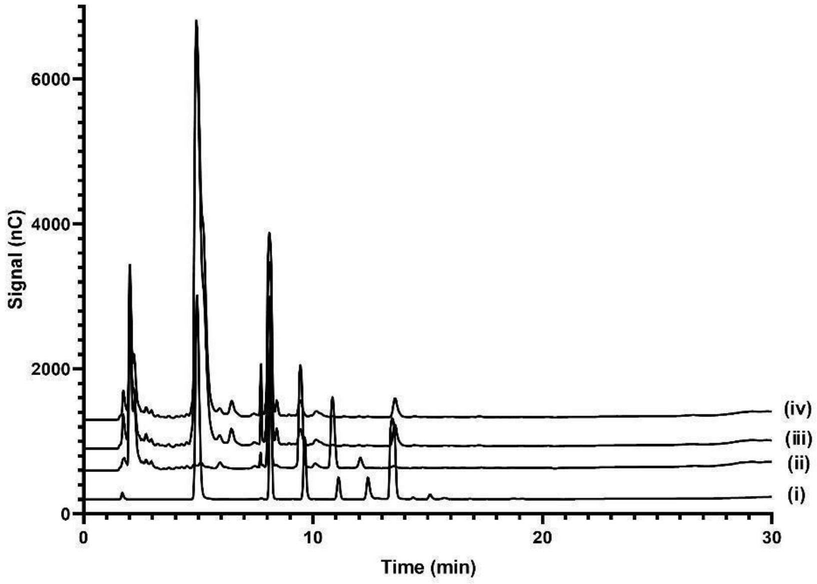

6000
4000
2000
0
Signal (nC)
0
10
20
30
Time (min)
(iv)
(iii)
(ii)
(i)


(c)

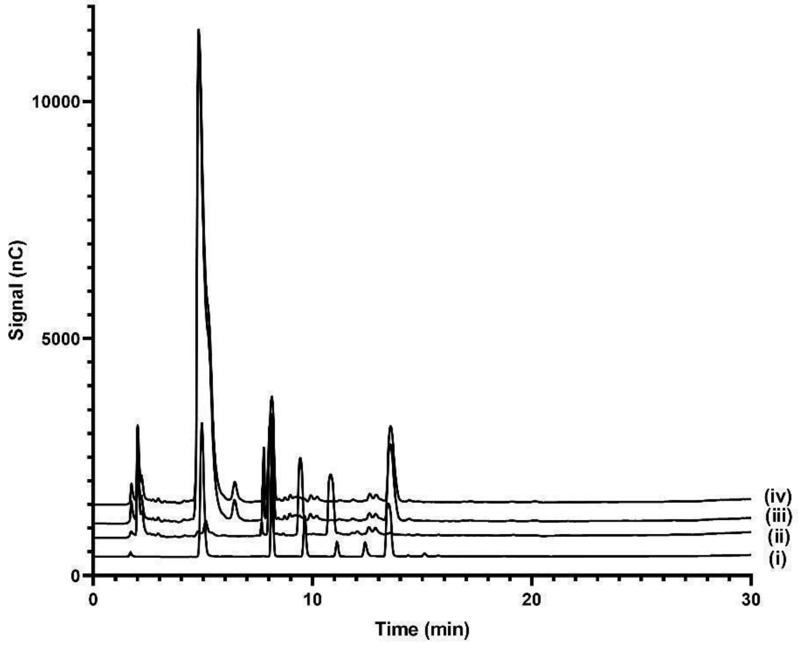

10000
5000
0
Signal (nC)
0
10
20
30
Time (min)
(iv)
(iii)
(ii)
(i)

**Figure S2:** *HPAEC-PAD chromatograms of the P. pulmonarius culture supernatants reaction on PASC. (a) Xylose, (b) Beechwood, (c) Corn stover. (i) Cellooligomers standard, (glucose, cellobiose, cellotriose, cellotetraose, cellopentaose, cellohexaose), (ii) reaction blind, without the addition of any supernatant, (iii)-(iv) reaction duplicates.*

**References**


(1) Bakouli, D. P.; Pedi, E.; Labrou, N.; Topakas, E.; Zerva, A. A Novel GH12 Xyloglucanase from the White Rot Fungus *Abortiporus Biennis*, Synergistically Enhances Lignocellulose Saccharification by Commercial Cellulases. *Enzyme Microb. Technol.* **2025**, *187*, 110628. https://doi.org/10.1016/j.enzmictec.2025.110628.

(2) Katsimpouras, C.; Kalogiannis, K. G.; Kalogianni, A.; Lappas, A. A.; Topakas, E. Production of High Concentrated Cellulosic Ethanol by Acetone/Water Oxidized Pretreated Beech Wood. *Biotechnol. Biofuels* **2017**, *10* (1), 54. https://doi.org/10.1186/s13068-017-0737-9.

(3) Gabriel, L.; Brůna, T.; Hoff, K. J.; Ebel, M.; Lomsadze, A.; Borodovsky, M.; Stanke, M. BRAKER3: Fully Automated Genome Annotation Using RNA-Seq and Protein Evidence with GeneMark-ETP, AUGUSTUS, and TSEBRA. *Genome Res.* **2024**, *34* (5), 769–777. https://doi.org/10.1101/gr.278090.123.

(4) Gabriel, L.; Hoff, K. J.; Brůna, T.; Borodovsky, M.; Stanke, M. TSEBRA: Transcript Selector for BRAKER. *BMC Bioinformatics* **2021**, *22* (1), 566. https://doi.org/10.1186/s12859-021-04482-0.

(5) Holt, C.; Yandell, M. MAKER2: An Annotation Pipeline and Genome-Database Management Tool for Second-Generation Genome Projects. *BMC Bioinformatics* **2011**, *12* (1). https://doi.org/10.1186/1471-2105-12-491.

(6) Haas, B. J.; Zeng, Q.; Pearson, M. D.; Cuomo, C. A.; Wortman, J. R. Approaches to Fungal Genome Annotation. *Mycology* **2011**, *2* (3), 118–141. https://doi.org/10.1080/21501203.2011.606851.

(7) Zheng, J.; Ge, Q.; Yan, Y.; Zhang, X.; Huang, L.; Yin, Y. dbCAN3: Automated Carbohydrate-Active Enzyme and Substrate Annotation. *Nucleic Acids Res.* **2023**, *51* (W1), W115–W121. https://doi.org/10.1093/nar/gkad328.

(8) Li, J.; Han, L.-H.; Liu, X.-B.; Zhao, Z.-W.; Yang, Z. L. The Saprotrophic *Pleurotus Ostreatus* Species Complex: Late Eocene Origin in East Asia, Multiple Dispersal, and Complex Speciation. *IMA*

*Fungus* **2020**, *11* (1), 10. https://doi.org/10.1186/s43008-020-00031-1.

(9) Katoh, K.; Standley, D. M. MAFFT Multiple Sequence Alignment Software Version 7: Improvements in Performance and Usability. *Mol. Biol. Evol.* **2013**, *30* (4), 772–780. https://doi.org/10.1093/molbev/mst010.

(10) *ClipKIT: A multiple sequence alignment trimming software for accurate phylogenomic inference | PLOS Biology*. https://journals.plos.org/plosbiology/article?id=10.1371/journal.pbio.3001007 (accessed 2025-05-27).

(11) Minh, B. Q.; Schmidt, H. A.; Chernomor, O.; Schrempf, D.; Woodhams, M. D.; Haeseler, A. von; Lanfear, R. IQ-TREE 2: New Models and Efficient Methods for Phylogenetic Inference in the Genomic Era. *Mol. Biol. Evol.* **2020**, *37* (5), 1530–1534. https://doi.org/10.1093/molbev/msaa015.

(12) Yu, G.; Smith, D. K.; Zhu, H.; Guan, Y.; Lam, T. T.-Y. Ggtree: An r Package for Visualization and Annotation of Phylogenetic Trees with Their Covariates and Other Associated Data. *Methods Ecol. Evol.* **2017**, *8* (1), 28–36. https://doi.org/10.1111/2041-210X.12628.

(13) Zerva, A.; Siaperas, R.; Taxeidis, G.; Kyriakidi, M.; Vouyiouka, S.; Zervakis, G. I.; Topakas, E. Investigation of *Abortiporus Biennis* Lignocellulolytic Toolbox, and the Role of Laccases in Polystyrene Degradation. *Chemosphere* **2023**, *312*, 137338. https://doi.org/10.1016/j.chemosphere.2022.137338.

(14) May, D. H.; Tamura, K.; Noble, W. S. Param-Medic: A Tool for Improving MS/MS Database Search Yield by Optimizing Parameter Settings. *J. Proteome Res.* **2017**, *16* (4), 1817–1824. https://doi.org/10.1021/acs.jproteome.7b00028.

(15) Kong, A. T.; Leprevost, F. V.; Avtonomov, D. M.; Mellacheruvu, D.; Nesvizhskii, A. I. MSFragger: Ultrafast and Comprehensive Peptide Identification in Mass Spectrometry–Based Proteomics. *Nat. Methods* **2017**, *14* (5), 513–520. https://doi.org/10.1038/nmeth.4256.

(16) Geiszler, D. J.; Kong, A. T.; Avtonomov, D. M.; Yu, F.; Leprevost, F. da V.; Nesvizhskii, A. I. PTM-Shepherd: Analysis and Summarization of Post-Translational and Chemical Modifications From

Open Search Results. *Mol. Cell. Proteomics* **2021**, *20*, 100018. https://doi.org/10.1074/mcp.TIR120.002216.

(17) Giorgi, F. M.; Bolger, A. M.; Lohse, M.; Usadel, B. Algorithm-Driven Artifacts in Median Polish Summarization of Microarray Data. *BMC Bioinformatics* **2010**, *11* (1), 553. https://doi.org/10.1186/1471-2105-11-553.

(18) Nygaard, V.; Rødland, E. A.; Hovig, E. Methods That Remove Batch Effects While Retaining Group Differences May Lead to Exaggerated Confidence in Downstream Analyses. *Biostat. Oxf. Engl.* **2016**, *17* (1), 29–39. https://doi.org/10.1093/biostatistics/kxv027.

(19) Zerva, A.; Pentari, C.; Termentzi, A.; America, A. H. P.; Zouraris, D.; Bhattacharya, S. K.; Karantonis, A.; Zervakis, G. I.; Topakas, E. Discovery of Two Novel Laccase-like Multicopper Oxidases from *Pleurotus Citrinopileatus* and Their Application in Phenolic Oligomer Synthesis. *Biotechnol. Biofuels* **2021**, *14* (1), 83. https://doi.org/10.1186/s13068-021-01937-7.

(20) Chorozian, K.; Karnaouri, A.; Tryfona, T.; Kondyli, N. G.; Karantonis, A.; Topakas, E. Characterization of a Novel AA16 Lytic Polysaccharide Monooxygenase from *Thermothelomyces Thermophilus* and Comparison of Biochemical Properties with an LPMO from AA9 Family. *Carbohydr. Polym.* **2024**, *342*, 122387. https://doi.org/10.1016/j.carbpol.2024.122387.

(21) Miller, G. L. Use of Dinitrosalicylic Acid Reagent for Determination of Reducing Sugar. *Anal. Chem.* **1959**, *31* (3), 426–428. https://doi.org/10.1021/ac60147a030.
